# The Mechanism and Pathways of Dopamine and Dopamine Agonists in Prolactinomas

**DOI:** 10.3389/fendo.2018.00768

**Published:** 2019-01-22

**Authors:** Xiaoshuang Liu, Chao Tang, Guodao Wen, Chunyu Zhong, Jin Yang, Junhao Zhu, Chiyuan Ma

**Affiliations:** ^1^The State Key Laboratory of Pharmaceutical Biotechnology and Jiangsu Engineering Research Center for MicroRNA Biology and Biotechnology, School of Life Science, NJU Advanced Institute for Life Sciences, Nanjing University, Nanjing, China; ^2^Department of Neurosurgery, School of Medicine, Jinling Hospital, Nanjing University, Nanjing, China; ^3^Tungwah Hospital of Sun Yat-Sen University, Dongguan, China; ^4^School of Medicine, Nanjing Medical University, Nanjing, China

**Keywords:** dopamine agonists, bromocriptine, cabergoline, programmed cell death, prolactinomas

## Abstract

Dopamine agonists such as bromocriptine and cabergoline are the predominant treatment drugs for prolactinoma by inhibiting prolactin secretion and shrinking tumor size. However, the pathways of either dopamine or its agonists that lead to the death of cells are incompletely understood and some are even conflicting conclusions. The main aim of this paper is to review the different pathways of dopamine and its agonists in prolactinomas to help to gain a better understanding of their functions and drug resistance mechanisms.

## Introduction

Pituitary adenomas (PAs) are common intracranial neoplasms. Typically, PAs are classified as either clinically non-functioning PAs or functioning PAs with characteristic clinical and endocrine symptoms, such as acromegaly and hyperprolactinemia or Cushing disease (1, 2).

Prolactinomas are the most common type of functioning PAs, which can cause headache, visual dysfunction, hypopituitarism, and hyperprolactinemia (3). The clinical features of hyperprolactinemia include impotence in males and oligo/amenorrhea in females (4, 5). The normalization of serum prolactin (PRL) levels and shrinkage of tumors are among the major goals of treatment in patients with prolactinomas (6). Dopamine agonists (DAs), such as bromocriptine (BRC) and cabergoline (CAB) are the first-line drugs for the treatment of patients with idiopathic hyperprolactinemia and prolactinomas (3, 7). The lactotroph adenoma cells express dopamine receptors, and DAs effectively suppress prolactin secretion and shrink the tumor by binding the cell-surface dopamine receptors in most patients (7, 8). This suggests that a “gene-network” may exist to regulate the activation of dopamine receptors, and may be involved in the mechanism of action of DAs for the treatment prolactinomas.

Although, two main DAs, namely BRC and CAB, have been approved as first-line drugs for the treatment of patients with hyperprolactinemia, a minority of patients with prolactinoma were resistant or intolerant to BRC, but responded adequately to CAB (9, 10). Currently, a better understanding of the pathophysiology of prolactinomas and the precise mechanisms of action of DAs in prolactinomas is greatly needed, especially considering that different pharmacological compounds act on lactotroph cells through different intracellular molecular pathways.

**Figure 1:**
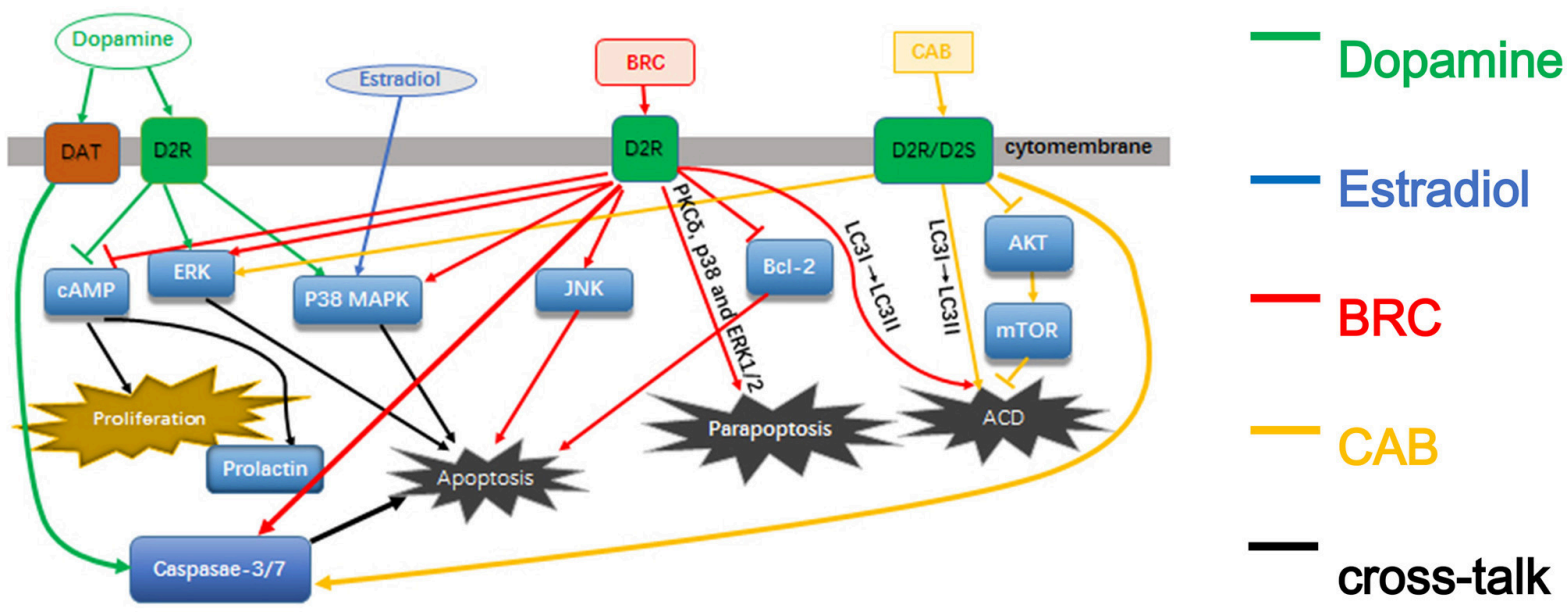
The pathways of dopamine and dopamine agonists in prolactinomas.

In this review, we summarize the current research advances on different pathways and mechanisms of dopamine and DAs effects on prolactinoma cells to help accelerate future research in this field (Figure 1).

## Dopamine and Dopamine Receptors

Multiple *in vitro* and *in vivo* studies have demonstrated that dopamine is an effective inhibitor of PRL secretion (11, 12), PRL gene expression and lactotroph cell proliferation (13). It can also induces the apoptosis of lactotroph cells (14).

Based on the functions of the dopamine receptors, they can be divided into D1-like receptors, such as D1 and D5, and D2-like receptors including D2, D3, and D4. The two DA receptor families play different roles. For example, D1-like receptors can induce the production of cyclic adenosine monophosphate (cAMP) and activate cAMP-dependent protein kinase (PKA) (15). Conversely, D2-like receptors (D2, D3, and D4) can reduce the accumulation of cAMP through interaction with Gi/G0 proteins (16). The activation of D2 receptors can also inhibit PRL secretion by decreasing the cell calcium levels through the G13 protein (17), but the activation of D1 receptors instead stimulates PRL secretion by stimulating vasoactive intestinal peptide (VIP) secretion (18, 19).

There are two isoforms of D2R produced by alternative splicing, namely the short and long isoforms (D2S and D2L) (13), which differ by only 29 amino acids derived from an additional exon in D2L, encoding the third intracellular loop of the receptor (20). D2S and D2L receptors are hypothesized to have distinct functions in the mitogen-activated protein kinase (MAPK) pathways (21). The pituitary size and PRL levels were found to be reduced in mice overexpressing D2S compared to wild type (WT) or D2L overexpressing mice (22). These observations suggest that dopamine effects on lactotrophs are mediated through the D2S receptor isoform and is an estrogen-dependent process. The decrease of D2S expression may play a part in D2R agonist resistant prolactinomas (21). In the pituitary gland, the expression level of D2L is much lower than that of D2S (20).

Most researchers use rodent or murine tumor cell lines to study dopamine functions in the pituitary and PAs (22, 23). In particular, studies on the rodent GH3 pituitary cell line have contributed significantly to the understanding of mechanisms of dopamine-induced apoptosis (23, 24). The receptors for VIP, thyroid-stimulating hormone (TRH) were found in GH3 cells, but no dopamine receptors (25). Many studies have demonstrated that GH3 cells do not express functional D2 receptors (26, 27). Indeed, some studies suggested that dopamine-induced apoptosis could not occur in the GH3 cell line unless it was transfected with a functional D2R (26).

## Dopamine Reduce PRL and Induce Apoptosis of Pituitary Adenoma Cells

In cells expressing either transfected or endogenous D2R receptors, the p38 MAPK or extracellular-signal-regulated kinase (ERK) were shown to be involved in the process of dopamine-induced apoptosis (22, 26). However, it should be noted that there are many conflicting reports about the regulation of the ERK pathway by the D2S receptor and it could be a cell type-dependent process. Previous research found that in non-neuronal cells, dopamine-D2 receptors stimulate ERK activity and cell proliferation (28). However, in neuroendocrine cells, such as GH4-rD2S, the phosphorylation of ERK was inhibited by D2S receptors (29). Another study found that in normal rat pituitary cells, ERK was inhibited by D2R (30). There is another hypothesis suggesting that the regulation of the ERK pathway by dopamine is a dynamic process, whereby the activated ERK may be reduced by dopamine to antagonize the stimulation thus leading to changes in gene expression and cell growth (30).

Different from these findings, another study demonstrated that the apoptosis induced by dopamine is promoted through the dopamine transporter (DAT) instead of D2R (23). In contrast, based on this assumption, in a co-culture experiments with a specific DAT inhibitor and dopamine, the apoptotic response was not attenuated, thus indicating that dopamine-induced apoptosis is not mediated through the DAT (31). Nevertheless, in GH3 cells which do not express D2R, an increase in apoptosis was observed with increasing time and concentration of dopamine (23, 31). Although no activation of any of the analyzed MAPKs was observed within 0.25–24 h, including p38-kinase, JNK, and ERK which is different from BRC challenged cells (23, 31). These observations indicate that dopamine may also induce apoptosis through other receptors and pathways.

Some studies indicated that the apoptosis of lactotrophs induced by dopamine is also an estrogen-dependent process (21). Studies on PRL cells found that it is not sufficient for D2S to induce apoptosis by dopamine, and estradiol-dependent activity is also needed. Estradiol can also increase the phosphorylation of p38 MAPK induced by dopamine. Despite this, the phosphorylation of p38 is induced by D2S activation regardless of the presence or absence of estradiol (21). Based on these findings, estradiol seems to be necessary but not sufficient for p38 MAPK phosphorylation to induce apoptosis in lactotrophs. The expression of p53 was also found to be increased by estradiol in anterior pituitary cells (32). As p53 is a target of p38 MAPK, the estradiol on anterior pituitary cells may induce p53 activation by increasing p38 MAPK phosphorylation (33).

## Dopamine Agonists

BRC and CAB are the two main DAs used as first-line treatment for prolactinomas, including microprolactinomas, macroprolactinomas, and giant prolactinomas. They can inhibit PRL secretion and shrink tumors effectively.

BRC was the first dopamine agonist used in clinical practice. It is a D2 receptor agonist, as well as D1 receptors antagonist. BRC is a semi-synthetic ergot derivative which binds to the D2R of anterior pituitary cells, especially on lactotrophs. The secretion of PRL is decreased by BRC through the stimulation of Na^+^, K^+^-ATPase activity and/or cytosolic Ca^2+^ elevation, which further inhibit the production of cAMP (34).

CAB has a higher affinity and selectivity for D2 receptors compared with BRC. In most people, CAB is more effective and has a longer half-life than BRC. It is also better-tolerated and has fewer side effects. For patients who are resistant or not very responsive to BRC, CAB has been proven to be effective (10). Besides prolactinomas, CAB is also effective for other types of PAs, such as acromegalic and ACTH-secreting adenomas (35). Accordingly, it is a valuable medicine for PAs.

The effect of CAB on reducing the size of prolactinomas is also mediated through the activation of the D2R (D2S) of anterior pituitary cells and is estrogen-dependent. Studies have found that ERK and phosphatidylinositol 3-kinase (PI3K) signaling is oppositely regulated by D2S and D2L, with D2L inhibiting both pathways, and D2S stimulating both pathways once activated by CAB (36).

### DAs Induced Pituitary Adenoma Cell Death

According to different criteria, such as morphological appearance, immunological characteristics, enzymological property and functions (37), programmed cell death (PCD) can be classified into three main types (38). Type 1 is known as apoptosis, in which cells display obvious morphological appearance, such as cytoplasmic, nuclear shrinkage, and chromosomal DNA fragmentation. The activation of caspases is a central mechanism of apoptosis (39). Type 2 corresponds to autophagic cell death (ACD), in which cells show regular degradation and recycling of cellular components (40). Mammalian target of rapamycin (mTOR) and PI3K pathways are considered as primary autophagy regulatory pathways (41). Microtubule-associated protein 1A/1B-light chain 3 (LC3) is also associated with autophagy activation. A cytosolic form of LC3 (LC3-I) is converted to an LC3-phosphatidylethanolamine conjugate (LC3-II), which is associated with autophagic vesicles (42). Type 3 is called paraptosis, which is a non-lysosomal vacuolated degeneration (43, 44). The features of paraptosis are cytoplasmic vacuoles, lack of apoptotic morphology and independence of caspase activation and inhibition (45).

#### BRC Induces Apoptosis

During treatment with BRC, typical apoptotic features were found in GH3 and AtT-20 cell lines, such as fragmented nuclei and condensed chromatin, which are indicative of apoptosis (46). The proportion of tumor cells undergoing apoptosis also increased with time (46). As an initial anti-apoptotic regulator, which protect cells from apoptosis (47), the suppression of bcl-2 was also observed in BRC-treated GH3 cells and AtT-20 cells (46).

Studies in GH3 cells revealed that apoptosis induced by BRC is regulated through the activation of certain MAPK pathway members, such as p38-MAPK, JNK, and ERK (24, 31). P38 MAPK was found to be more closely associated with BRC-induced apoptosis. However, inhibition of p38 MAPK did not reduce the apoptotic effect of BRC (31). Accordingly, there may be other mechanisms mediating the apoptotic response to BRC and they should be studied to understand such a complex regulatory process involving numerous factors (24, 46).

Additional studies in GH3 cells show that dopamine and BRC utilize distinct intracellular pathways. BRC-induced apoptosis is sensitive to the inhibition of JNK, whereas dopamine-induced apoptosis is not. However, subsequently caspase-3/7 can be activated by both of them (31). The activation of JNK precedes cytochrome c release (31). In dopamine-treated cells the release of mitochondrial cytochrome c was also observed but it was preceded by an increase in reactive oxygen species (ROS) (23, 31). Through engagement and co-activation of these pathways BRC and dopamine ultimately synergistically induce cell death (31). These findings have motivated us to study the effects of these drugs in co-incubation experiments (31).

#### CAB Induces Apoptosis

The CAB-induced apoptosis observed in PRL-D2S cells involved the p38 MAPK pathways and could be reverted by a p38 MAPK inhibitor (21). Another study in MMQ cells, a prolactin-secreting clonal cell line that is responsive to dopamine, demonstrated that CAB increased the expression of apoptotic related proteins, such as PARP and cleaved caspase-3, indicating that the apoptosis is caspase-dependent (42). However, in CAB-treated GH3 cells, the PARP protein was not involved in the process of cell death (42).

### DA-Regulated Paraptosis and ACD

Some studies have demonstrated that CAB and BRC not only induce apoptosis but also non-apoptotic cell death (42, 48). The autophagic degradation of organelles which precedes nuclear destruction is an important characteristic of ACD (49). The JNK pathway may also participate in ACD (50). Cytoplasmic vacuolization in mitochondria and endoplasmic reticulum is one of the morphological features of paraptosis, and do not involve the lysosomal system (48). Paraptotic cells lack apoptotic morphology (48). For apoptosis, an explicit mechanism is the activation of caspases (39), but it is not involved in parapoptosis. ERK1/2 has also been shown to promote cell death by paraptosis in 293T cells and Hepa1c1c7 cells (45).

#### BRC Induces Parapoptosis and ACD

Protein kinase C δ (PKCδ) is also involved in tumor progression of various tumor types and plays an important role in the PCD of prolactinomas cells. A study in GH3B6 tumor somatolactotrophic cells found that PKCδ may also contribute to the apoptotic process (51). Also, a study on male rats found that BRC caused mainly paraptosis instead of apoptosis with the involvement of PKCδ, p38, and the ERK1/2 pathways. As indicated by the absence of morphological features of apoptosis, such as internucleosomal fragmentation and the production of an unspecific smear compatible with necrosis, as well as the failure to detect the active fragment of caspase 3 in the experiment (48).

It has also been reported that BRC may induce cell death by ACD, as indicated by a higher conversion ratio of LC3-I to LC3-II found in MMQ and GH3 cells compared with the controls (52). BRC could also regulate the cell cycle as more cells were arrested in the G_0_-G_1_ phase and there were much fewer cells in the S phase compared with the controls. However, the precise mechanism still remains to be elucidated (52). Several cell cycle regulators may be important for such study, such as cyclin E/D1, p16/21/27, etc. (5).

#### CAB Induces ACD

Several studies have demonstrated that autophagic and apoptotic cell death may coexist in CAB-mediated tumor shrinkage, as a result of the release of lysosomal enzymes (42, 53).

In MMQ and GH3 cells treated with CAB, a time-dependent decrease in mTOR and AKT phosphorylation was found, indicating that ACD is involved in CAB-treated cells through the inhibition of the mTOR or AKT pathways (42). In addition, it has been found that the AKT and mTOR pathways can regulate cell survival and death by integrating signals from various stresses and growth factors (54). mTOR has also been identified as a negative regulator of ACD (55). The conversion of LC3-I to LC3-II was also detected in GH3 and MMQ cells at early stages of CAB treatment (42). By knocking-down certain proteins, such Becn1 and ATG5/7, which are essential for autophagy, it was confirmed that CAB can induce ACD (42).

## Discussion

Prolactinomas are the most common pituitary tumors and DAs have been shown to be highly effective in most cases. Nevertheless, many patients, who do not respond satisfactorily to DAs, are considered to be drug resistant (56, 57). The potential mechanisms involved in such resistance are not completely understood. Some studies found that less D2R mRNA was expressed in prolactinomas patients who are resistant to DAs compared to responsive patients (58). As another key receptor in prolactinomas, the estrogen receptor also plays important roles in tumorigenesis, metastasis and therapy (59), which should be studied further. Some studies have found that DA-induced apoptosis in lactotrophs is an estrogen and D2R dependent process. Furthermore, in DA-resistant prolactinoma patients, the D2L/D2S expression ratio has been found to be reduced (60), which is contradictory to other studies (21, 61, 62). Noteworthy, some studies found that the expression of D2S mRNA was significantly different for invasive and non-invasive tumors (62), thus researchers should pay more attention to the patients/cell lines in the studies. Since D2L and D2S receptors have distinct functions in MAPK pathways, more studies should be focused on them, especially in cell lines transfected with D2R. Reduced TGFβ1 activity is a common feature in the development of prolactinoma, studies also found that the recovery of TGFβ1 activity emerges as a novel therapeutic target for the treatment of DA-resistant patients (6). According to some studies, in diabetic patients with different types of tumors, metformin showed a survival benefit (63). There were two clinical cases showing that the combination of BRC and metformin might be a new effective therapy for DA-resistant prolactinomas patients (64). Ultimately, there is a great need to explore the molecular mechanisms of dopamine and DAs effects on prolactinomas in order to find a better treatment.

It has been confirmed by many studies that apoptosis induced by DAs is mediated through D2S, involve the activation of the MAPK pathway and is an estrogen-dependent process. However, studies in cell lines without dopamine receptors, such as GH3, indicate that DAs can also induce apoptosis without the activation of any of the MAPKs, suggesting that other receptors may participate in the process. In BRC-treated GH3 cells, which do not have the D2R, apoptosis is induced and is closely associated with the activation of p38 MAPK. However, the inhibition of p38 MAPK has no impact on the apoptotic response, so other mechanisms may contribute to the apoptotic process, which need to be explored.

Although some studies have demonstrated the involvement of paraptosis or autophagic mode of cell death in BRC and CAB treated cells, more evidence is still needed. Also, even though CAB and BRC are both dopamine agonists, the signal transduction pathways activated by the two drugs seem to be different. It has been found that the inhibition of p38 MAPK can revert CAB-induced apoptosis, which is different from BRC. Autophagy and apoptosis are also considered to coexist in CAB-treated cells. Autophagy, paraptosis and apoptosis are different cell death modes that share some regulators, thus further studies should be concentrated on the detailed regulation of DAs in prolactinoma. Finally, dopamine-induced oxidative stress has been proposed as a potential mechanism of apoptosis and neurotoxicity (65). Since it has been reported that dopamine neurotoxicity can induce the death of neurons (66), more attention should be paid to the cytotoxic mechanisms of dopamine in pituitary adenoma cells.

## Author Contributions

GW, CZ, JY, and JZ carried out the literature search. CT and XL drafted the manuscript. CT and CM performed manuscript review.

### Conflict of Interest Statement

The authors declare that the research was conducted in the absence of any commercial or financial relationships that could be construed as a potential conflict of interest.
